# Ubiquitin Carboxy-Terminal HydrolaseL3 Correlates with Human Sperm Count, Motility and Fertilization

**DOI:** 10.1371/journal.pone.0165198

**Published:** 2016-10-25

**Authors:** Meijiao Wang, Tinghe Yu, Lina Hu, Zhi Cheng, Min Li

**Affiliations:** 1 Department of Physiology, West China School of Preclinical and Forensic Medicine, Sichuan University, Chengdu, China; 2 Key Medical Laboratory of Obstetrics and Gynecology, The Second Affiliated Hospital, Chongqing Medical University, Chongqing, China; 3 Center of Reproductive Medicine of Obstetrics and Gynecology, The Second Affiliated Hospital, Chongqing Medical University, Chongqing, China; 4 School of Basic Medical Sciences, Chongqing Medical University, Chongqing, China; Universite Blaise Pascal, FRANCE

## Abstract

Ubiquitin C-terminal hydrolase L3 (UCHL3) belongs to the group of deubiquitinating enzymes and plays a part in apoptosis of germ cells and the differentiation of spermatocytes into spermatids. However, the exact role of UCHL3 in human spermatogenesis and sperm function remains unknown. Here we examined the level and activity of UCHL3 in spermatozoa from men with asthenozoospermia (A), oligoasthenozoospermia (OA) or normozoospermia (N). Immunofluorescence indicated that UCHL3 was mainly localized in the acrosome and throughout the flagella, and western blotting revealed a lower level in A or OA compared with N (*p* < 0.05). The catalytic activity of UCHL3 was decreased in spermatozoa from A or OA (*p* < 0.05, *p* < 0.001, respectively). The level and activity of UCHL3 were positively correlated with sperm count, concentration and motility. The UCHL3 level was positively correlated with the normal fertilization rate (FR) and percentage of embryos suitable for transfer/cryopreservation of *in vitro* fertilization (IVF). The UCHL3 activity was also positively correlated with FR, the percentage of embryos suitable for transfer/cryopreservation and high-quality embryos rate of IVF. Aforementioned correlations were not manifested in intra-cytoplasmic sperm injection (ICSI). These findings suggest that UCHL3 may play a role in male infertility.

## Introduction

Male infertility is a worldwide medical problem with increasing incidence, and the etiology is multifactorial. More than 80% of semen samples from infertile men show poor sperm motility (asthenozoospermia, A) and/or low sperm concentration (oligoasthenozoospermia, OA) [1]. Most infertile men can actually produce spermatozoa [2], but they are incapable of fertilizing an oocyte naturally. Indeed, sperm motility, morphology and function of the acrosome, which are established during spermiogenesis, are determinants of the critical process of fertilization [3].

Ubiquitin-proteasome system (UPS) is necessary for all steps of mammalian spermatogenesis and fertilization [4–7]. However, the role of UPS in spermiogenesis and male infertility is poorly understood. Ubiquitin molecules are covalently attached to target proteins by a series of enzymes, including ubiquitin activating enzyme (E1), ubiquitin-conjugating enzyme (E2) and ubiquitin ligase (E3). Ubiquitinated proteins are usually degraded by the 26S proteasome or lysosomes, and monomeric ubiquitin is released from the polyubiquitin chain or a substrate protein through the action of deubiquitinating enzymes (DUBs) [8]. DUBs play an important role in regulation of spermatogenesis [9]. There are two classes of sperm DUBs: ubiquitin C-terminal hydrolases (UCHs) and ubiquitin-specific proteases (USPs) [9–11].

UCHs, as an important regulator of UPS and a small molecular-mass cysteine protease, can remove the short/flexible peptide chain from the carboxy-terminus of ubiquitin [12]. UCHs members (i.e., UCHL3, UCHL2, UCHL4, UCHL5, and CYLD) are expressed during each stage of spermatogenesis and are involved in gonocyte recruitment, cell-cycle progression, meiotic-phase progression, spermiogenesis, the formation of high quality sperm and germ-cell apoptosis [7, 9, 13]. Of those five members in the UCH family, UCHL3 and UCHL1 are dominant [14]. UCHL3 is expressed ubiquitously in all tissues, with a high level in the testis (including spermatocytes and spermatids) [14, 15]. Previous studies have demonstrated that UCHL3 is involved in many physiological functions, including reproduction. UCHL3 may play a role in apoptosis of germ cells and the meiotic differentiation of spermatocytes into spermatids [15, 16]. However, the detailed role of UCHL3 in spermatogenesis has not yet been elucidated.

In this study, semen samples from subjects undergoing *in vitro* fertilization (IVF) or intra-cytoplasmic sperm injection (ICSI) were analyzed, and the localization, level and enzymatic activity of UCHL3 in spermatozoa were determined. Correlations between UCHL3 and sperm count, concentration, motility, fertilization, and embryo quality were evaluated. The findings indicated that UCHL3 can be an indicator of sperm quality in A and OA.

## Materials and Methods

### Semen sample collection and preparation

The study was approved by the Ethics Committee of the Second Affiliated Hospital of Chongqing Medical University, and signed informed consent was obtained from all participants (reference number2013–019; date of approval: 5 March, 2013).

Semen samples were collected from 92 subjects (aged 25–35 years) who received ICSI/IVF at the Reproductive Center of the aforementioned hospital between July 2013 and August 2015. Infertility was a result of defects in the oviduct (e.g., bilateral tubal obstruction, hydrosalpinx, tubal ligation and resection of bilateral tubes) and/or male factors (i.e., A and OA). All female partners with hysteromyoma, adenomyosis, ovarian tumor, endometriosis, polycystic ovarian syndrome, hyperprolactinemia, thyroid and adrenal diseases, diabetes or chromosomal aberration were excluded.

Semen specimens were obtained by masturbation after 3–7 days of abstinence. After liquefaction at 37°C for 30 min, semen samples were analyzed according to the World Health Organization(WHO) Standards [17]; sperm count, sperm concentration, total motility (TM), progressive motility (PR), and morphology were determined. Semen samples were identified as normozoospermia (N, *n* = 45), A (*n* = 29) and OA (*n* = 18; Table 1).

**Table 1:** Description of the semen parameters of subjects included in this study (mean ± SD).

Subject parameters	N (n = 45)	A (n = 29)	OA (n = 18)
**Sperm count (×10**^**6**^**)**	242.0 ± 96.8	122.3 ± 69.5	19.4 ± 11.4
**Sperm concentration (×10**^**6**^**/ml)**	69.9 ± 10.9	46.2 ± 18.2	7.8 ± 3.0
**Total motility (%)**	85.3 ± 3.5	44.1 ± 7.0	34.2 ± 13.0
**Progressive motility (%)**	51.9 ± 8.7	11.9 ± 7.2	9.7 ± 6.8
**Normal morphology (%)**	7.0 ± 1.5	3.4 ± 1.9	2.8 ± 1.7
**Age (years)**	30.6 ± 3.1	31.2 ± 3.3	30.6 ± 3.1
**Abstinence(days)**	4.9 ± 1.3	5.0 ± 1.5	4.7 ± 1.6

N: normozoospermia; A: asthenozoospermia; OA: oligoasthenozoospermia.

### Detection of UCHL3 with immunofluorescence

Smeared semen was fixed in 100% acetone for 15 min at 4°C and stored at –80°C until use. After thawing and warming up to room temperature, the smears were permeabilised with 1% Triton X-100 in phosphate buffered saline (PBS), pH 7.4, for 15 min. After washing three times for 15min with washing buffer [0.2% Tween 20 and 1% inactivated normal goat serum (INGS) in PBS], 5% INGS in PBS was added to block nonspecific sites on the spermatozoa for 50min. Anti-UCHL3 rabbit polyclonal antibody (1:50, Bioss, Beijing, China) was added, and the slides were incubated at 4°C overnight. The antibody diluent was PBS containing 0.5% Tween 20 and 1% INGS. Slides were washed with washing buffer more than three times for 15 min, and then the Alexa Fluor 488-labeled goat anti-rabbit IgG secondary antibody (1:2,000; Molecular Probes, Invitrogen, Carlsbad, CA, USA) was added. Thereafter, slides were washed once with washing buffer (10 min) and twice with PBS (10 min). The nucleus was stained with anti-fade VECTASHIELD^®^ Mounting Medium, including DAPI (H-1200; Vector Laboratories Inc, Burlingame, CA, USA).

The negative control was performed in the same way by substituting non-immune rabbit serum for the primary antibody. Slides were observed under a confocal laser scanning microscope (Leica TCS-SP5, Leica Microsystems, Wetzlar, Germany).

### Detection of protein by western blotting

UCHL3 protein was analyzed by western blot [18]. 1.0 × 10^7^ spermatozoa were lysed in 200 μL cold lysis buffer (7 M urea, 2 M thiourea, 4% CHAPS, 65mM DTT, 1mM PMSF, and 1 × proteinase cocktail). The extracts (50 μg protein) were loaded into each lane. The primary antibodies used included anti-UCHL3 rabbit polyclonal antibody (1:500; Bioss, Beijing, China), and anti-α-tubulin rabbit monoclonal antibody (1:5,000; ab52866, Abcam Inc., Cambridge, MA, USA). The secondary antibody used was the HRP-labelled goat anti-rabbit IgG (1:5,000; 111-035-003; Jackson ImmunoResearch Laboratories Inc., WestGrove, PA, USA). The band density was quantified using the ImageJ software (rsbweb.nih.gov/ij/).

### Determination of UCH activity

UCH enzymatic activity of spermatozoa was determined according to the method of Yi *et al*. [19]. The spermatozoa were washed in assay buffer (50 mM HEPES, pH 7.5; 0.5 mM EDTA; 0.1 mg/ml BSA; 1 mM DL-DTT), and the concentration was adjusted to 5.0 × 10^6^ spermatozoa/ml. Spermatozoa were sonicated six times, using a JY92-2D ultrasonic cell pulverizer (60 W, 20% amplitude, 5 s per sonication), followed by centrifugation for 30 min at 5,000 × g. The supernatant was prepared for subsequent assays. Protein concentration was determined using the Bradford method [20]. All procedures were performed at 4°C.

Ubiquitin 7-amido-4-methylcoumarin (Ubiquitin-AMC) (Biomol, Enzo Life Sciences, Inc., Farmingdale, NY, USA) was used as the substrate. Aliquots (100 μl) of assay buffer, ubiquitin-AMC (with a final concentration of 1.25 μM) and spermatozoa extracts (with a final concentration of 0.2 mg/ml) were added to a 96-well plate (Corning Costar, NY, USA).

The positive control was 50 nM recombinant UCHL3 (Boston Biochem Inc., Cambridge, MA, USA), which replaced the spermatozoa extracts. For the negative control, 5μM ubiquitin aldehyde (Biomol, Enzo Life Sciences, Inc., Farmingdale, NY, USA), an inhibitor for UCHs, was added. The aliquots were incubated for 30 min at 39°C, and the activity was determined using fluorospectrophotometry. The excitation and emission wavelengths were 380 and 460 nm, respectively.

### Ovarian stimulation and oocyte collection

Ovarian stimulation was performed using a GnRH agonist long protocol [21]: Pituitary down-regulation was achieved with triptorelin 0.1 mg, qd (Decapeptyl; Ferring Pharmaceuticals, Kiel, Germany) from the prior mid-luteal phase. Once ovarian suppression was achieved, the dose was reduced to 0.05 mg until the day of human chorionic gonadotropin (HCG) administration. When sonography determined the absence of a dominant follicle and hormone levels were: follicle stimulating hormone (FSH) < 5 mIU/ml, luteinizing hormone (LH) < 5 mIU/ml, estrogen (E2) < 50 pg/ml and progesterone (P) < 0.9 ng/ml, recombinant human FSH (Gonal-F; Merck Serono, Geneva, Switzerland) administration was initiated with a daily dosage of 150−300 IU. Follicular growth was monitored by ultrasonography. Oocyte maturation was induced by the administration of 5,000‒10,000 IU HCG (Lizhu Company, China); the dose was modulated according to the mean diameter and the number of leading follicles and serum E2 level. Aspiration of the oocytes was performed 36 h following HCG injection.

### IVF and ICSI procedures

IVF and ICSI procedures were performed by the same embryologist. Spermatozoa were prepared by PureCeption^TM^ gradient centrifugation technique (SAGE, Pasadena, CA, USA). The retrieved oocytes were inseminated after 3–6 h, with about 10,000 spermatozoa per oocyte in a 10-μl droplet of modified HTF medium supplemented with 10% Quinn's human serum albumin (SAGE, Pasadena, CA, USA).

ICSI was performed 40–42 h after human chorionic gonadotropin injection. Cumulus cells were removed by pipetting the oocytes in modified HTF medium containing 80 IU/ml of hyaluronidase (H-3757; Sigma Chemical, St Louis, MO, USA). Denuded oocytes with a first polar body were selected for ICSI, which was performed 0–3 h after oocyte denudation.

Approximately 2 h after IVF insemination or immediately after ICSI, oocytes were cultured in Quinn’s Advantage Cleavage media (SAGE, Pasadena, CA, USA) at 37°C and 5% CO_2_ atmosphere.

### Embryo culture

Normal fertilization rate (FR) was evaluated 16–18 h following IVF or ICSI. The presence of two pronuclei (2PN) and two polar bodies indicated fertilization; the day of fertilization was set as Day 0. Zygotes with 2PN were cultured in Quinn’s embryo culture medium (SAGE, Pasadena, CA, USA) overlaid with approximately 2 mm of heavy paraffin oil (SAGE, Pasadena, CA, USA) at 37°C and 5% CO_2_. Embryos were graded and evaluated for suitability for transfer and cryopreservation at Day 2 or 3.

### Outcome measures

IVF FR was defined as the number of fertilized oocytes divided by the number of oocytes inseminated. Similarly, ICSI FR was calculated as the percentage of the number of oocytes with 2PN divided by the number of mature MII oocytes injected with one spermatozoon. Cleavage and embryo quality were observed on Days 2 and 3. Cleavage rate was defined as the number of blastomeres divided by the number of fertilized oocytes. High-quality embryo rate was defined as the number of Grade I and II embryos divided by the number of 2PN zygotes. The percentage of embryos suitable for transfer/cryopreservation was defined as the number of transferred and cryopreserved embryos divided by the number of 2PN embryos.

### Statistical analysis

All data were processed with the Prism software (GraphPad, San Diego, CA). Results were presented as mean ± standard derivation. All variables were checked for normal distribution before statistical analyses. For western blot and enzyme activity assays, all results were analyzed by one-way ANOVA followed by a Bonferroni post-test. Correlations between the level of UCHL3 and deubiquitinating activity, sperm count, concentration, motility, fertilization and embryo quality were evaluated by calculating Spearman’s correlation coefficient. *p*< 0.05 was considered statistically significant.

## Results

### UCHL3 located in the acrosome and tail of spermatozoa, with lower level in A and OA

**Fig 1:**
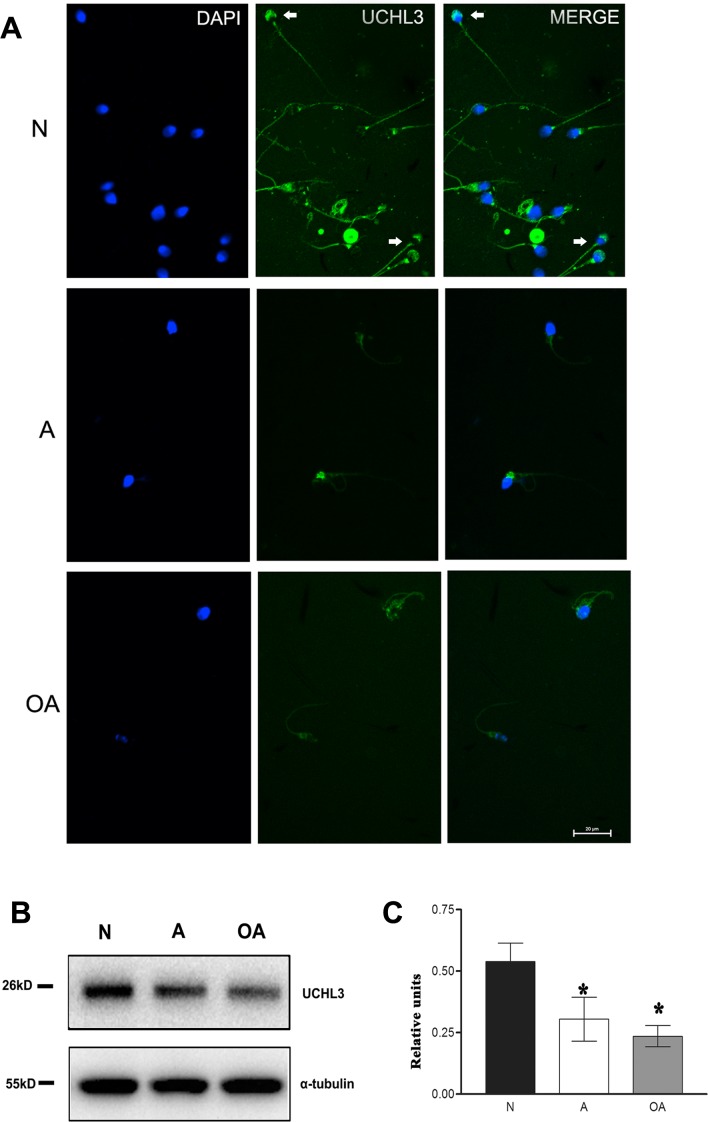
UCHL3 localization and level in asthenozoospermic and oligoasthenozoospermic men. (***A***) Representative immunofluorescence for the UCHL3 protein (green) in spermatozoa from normozoospermia, asthenozoospermia and oligoasthenozoospermia. UCHL3 mainly localized in the acrosome and the flagella (arrow). The scale bar was 20 μm. (***B*, *C***) Western blot detection for UCHL3. α-tubulin was the reference. Data were expressed as mean ± SD of three replicates.*p < 0.05 compared with N.

UCHL3 was mainly localized in acrosome and flagella (including the mitochondrial sheath) of spermatozoa from A or OA. There was also staining in the neck region of some spermatozoa. Spermatozoa from N subjects displayed a green staining in the normal acrosome and tail, whereas in spermatozoa from A or OA in the acrosomeless, small or abnormal acrosome and in the shorter or distorted tail. However, in the abnormal acrosome or distorted tail of spermatozoa from A or OA, UCHL3 levels were reduced or absent (Fig 1A).

Western blot demonstrated a lower level of UCHL3 in spermatozoa from A (0.57-fold of N) and OA (0.44-fold of N) in comparison with spermatozoa from N (Fig 1B and 1C; *p* < 0.05). These data indicated that UCHL3 was located in the acrosome and tail of a spermatozoon, with a lower level in spermatozoa from A or OA.

### UCHL3 level positively correlated with sperm count, concentration, total and progressive motility of sperm

**Fig 2:**
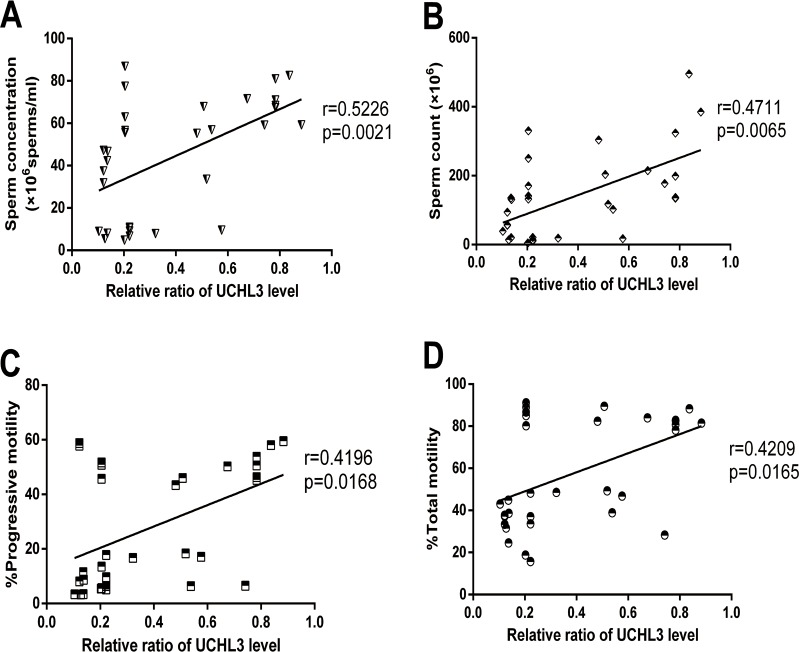
**Correlations of the UCHL3 level with** (***A***) sperm concentration, (***B***) sperm count and (***C***, ***D***) sperm motility (PR, TM) in spermatozoa from normozoospermia, asthenozoospermia, and oligoasthenozoospermia. Data were expressed as mean ± SD of three replicates.

Correlation analysis indicated that the UCHL3 level was positively correlated with most sperm quality parameters, including sperm count (*r* = 0.4711, *p* = 0.0065), sperm concentration (*r* = 0.5226, *p* = 0.0021), TM (*r* = 0.4209, *p* = 0.0165), and PR (*r* = 0.4196, *p* = 0.0168)(Fig 2).

### UCHL3 level positively correlated with the normal fertilization rate and percentage of embryos suitable for transfer/cryopreservation in IVF

**Fig 3:**
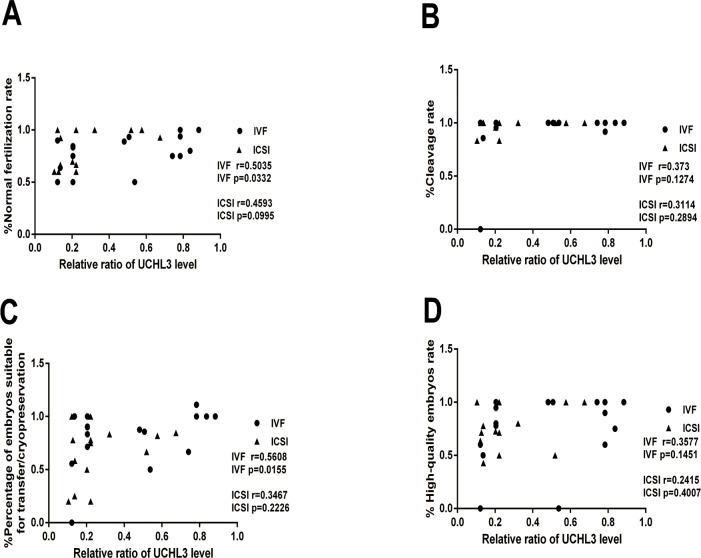
**Correlations of the UCHL3 level with** (***A***) Normal fertilization rate, (***B***) cleavage rate, (***C***) percentage of embryos suitable for transfer/cryopreservation and (***D***) high-quality embryo rate in spermatozoa from IVF and ICSI cycles. Data were expressed as mean ± SD of three replicates.

In IVF, the UCHL3 level was positively and linearly correlated with FR (*r* = 0.5035, *p* = 0.0332) and the percentage of embryos suitable for transfer/cryopreservation (*r* = 0.5608, *p* = 0.0155) (Fig 3A and 3C). However, in ICSI, no correlations were found between the UCHL3 level and FR (*r* = 0.4593, *p* = 0.0995), and the percentage of embryos suitable for transfer/cryopreservation (*r* = 0.3467, *p* = 0.2226). There was no correlation between the UCHL3 level and the cleavage rate (IVF: *r* = 0.373, *p* = 0.1274; ICSI: *r* = 0.3114, *p* = 0.2894), and the high-quality embryo rate (IVF: *r* = 0.3577, *p* = 0.1451; ICSI: *r* = 0.2415, *p* = 0.4007) in both IVF and ICSI (Fig 3).

### Spermatozoa from A or OA displayed a lower enzymatic activity of UCH

**Fig 4:**
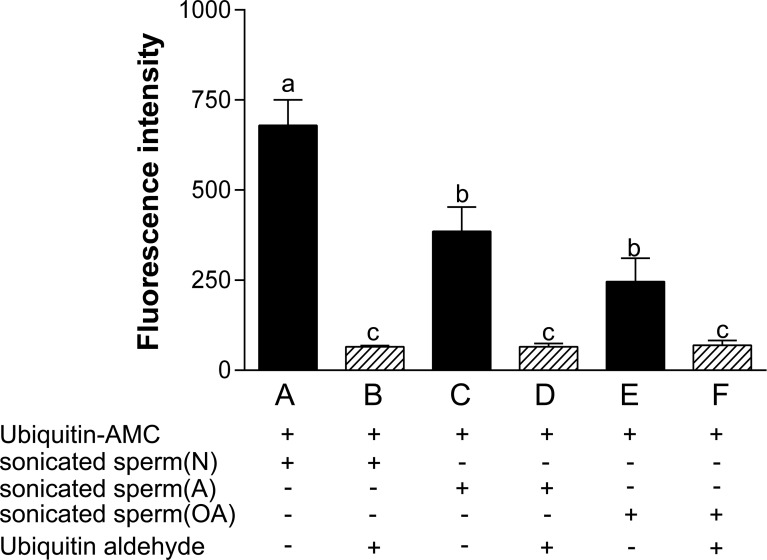
Enzymatic activity of ubiquitin C-terminal hydrolases was decreased in spermatozoa from asthenozoospermic and oligoasthenozoospermic men. Data were expressed as mean ± SD of three replicates. The inhibitor ubiquitin aldehyde was used as a negative control. Superscripts a, b and c denote significant differences at *p* < 0.05.

The enzymatic activity of ubiquitin C-terminal hydrolases in human spermatozoa from N, A and OA was measured. The UCH enzymatic activity in spermatozoa from A (0.57-fold of N; *p*<0.05) or OA (0.36-fold of N; *p*<0.001) was decreased in comparison with spermatozoa from N (Fig 4).

UCH activity in sperm extracts was reduced to a minimum by the specific UCH inhibitor, ubiquitin aldehyde (Fig 4).

### Enzymatic activity of UCH positively correlated with sperm count, concentration, total motility and progressive motility

**Fig 5:**
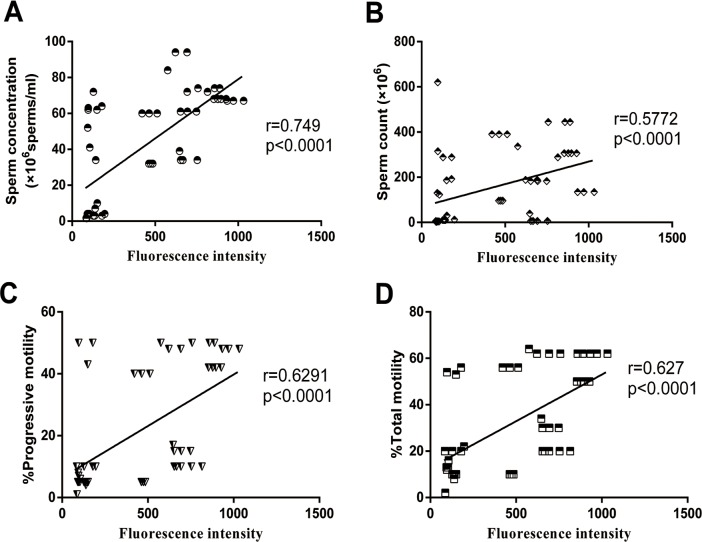
**Correlations of the UCH enzymatic activity with** (***A***) sperm concentration, (***B***) sperm count and (***C***, ***D***) sperm motility (PR, TM) in spermatozoa from normozoospermia, asthenozoospermia and oligoasthenozoospermia. Data were expressed as mean ± SD of three replicates.

Correlation analysis indicated that the UCH enzymatic activity was positivelyand linearly correlated with sperm count (*r* = 0.5772, *p* < 0.0001), sperm concentration (*r* = 0.749, *p* < 0.0001), TM (*r* = 0.627, *p* < 0.0001), and PR (*r* = 0.6291, *p* < 0.0001) (Fig 5).

### Enzymatic activity of UCH positively correlated with the normal fertilization rate, percentage of embryos suitable for transfer/cryopreservation and high-quality embryo rate in IVF

**Fig 6:**
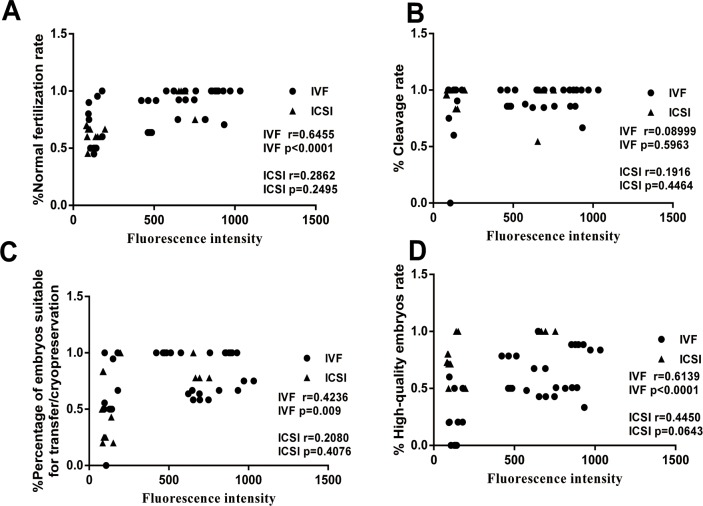
**Correlations of the UCH enzymatic activity with** (***A***) normal fertilization rate, (***B***) cleavage rate, (***C***) percentage of embryos suitable for transfer/cryopreservation and (***D***) high-quality embryo rate in spermatozoa from IVF and ICSI cycles. Data were expressed as mean ± SD of three replicates.

In IVF, the UCH enzymatic activity was positively and linearly correlated with FR (*r* = 0.6455, *p* < 0.0001), the percentage of embryos suitable for transfer/cryopreservation (*r* = 0.4236, *p* = 0.009) and high-quality embryo rate (*r* = 0.6139, *p* < 0.0001) (Fig 6A, 6C and 6D). However, no positive correlations were found between the UCH enzymatic activity and the FR (*r* = 0.2862, *p* = 0.2495), percentage of embryos suitable for transfer/cryopreservation (*r* = 0.2080, *p* = 0.4076), and high-quality embryo rate (*r* = 0.4450, *p* = 0.0643) in ICSI. The enzymatic activity did not correlate with cleavage rate in both IVF (*r* = 0.08999, *p* = 0.5963) and ICSI(*r* = 0.1916, *p* = 0.4464) (Fig 6).

## Discussion

UCHL3 mRNA was abundant in the mouse testis[14, 15], and this protein was present in testicular cells and mature spermatozoa of boars and the spermatocytes and spermatids of mice [15, 19]. However, the level and enzymatic activity of UCHL3 in spermatozoa from men with A or OA have not been validated yet. To address this question, we detected it by using immunofluorescence, western blotting, and activity assay.

UCHL3 was localized in the acrosome and throughout the whole flagella, including mitochondrial sheath, suggesting that UCHL3 can regulate fertilization and motility. This was consistent with the previous study showing that UCHL3 was present in the spermatid acrosomal cap and on the acrosome of boar spermatozoa, where it interacted with the surface of the zona pellucida and peripheral acrosomal plasma membrane proteins; UCHL3 may be involved in the acrosomal function and anti-polyspermy defense during porcine fertilization [19]. Western blotting analysis demonstrated a lower level of UCHL3 in spermatozoa in A and OA. Moreover, positive correlations were found among UCHL3 levels, sperm count, concentration, TM and PR. UCHL3 played a role in the prevention of mitochondrial oxidative stress-related apoptosis in photoreceptor cells [22]. An alteration in UCHL3 expression may affect both structure of the mitochondrial sheath and the level of reactive oxygen species (ROS), considering the role of mitochondria in the generation of ROS. ROS can affect the structural integrity of spermatozoa and their functions (i.e., sperm motility, capacitation, sperm–zona penetration, and sperm–oolemma fusion) [23]. The decrease in motility observed in this study suggested that UCHL3 may be associated with sperm motility. Furthermore, UCHL3 played a critical role in mediating apoptotic stress during spermatogenesis. Kwon *et al*. [24] showed that UCHL3 protected germ cells from apoptosis. Cryptorchid testes in *Uchl3* knockout mice showed a profound apoptotic germ cell loss and a slight increase of apoptotic proteins [16]. The reduction in expression of UCHL3 may affect anti-apoptotic function, thereby decreasing sperm count and concentration. The present data indicated that UCHL3 level was positively correlated with FR and the percentage of embryos suitable for transfer/cryopreservation during IVF. However, no correlations were confirmed between the UCHL3 level and FR and the percentage of embryos suitable for transfer/cryopreservation during ICSI, and the rates of cleavage and of high-quality embryos in both IVF and ICSI. Mtango *et al*. [25] found that the inhibition of UCHs caused a reduction in FR, abnormal fertilization, and failure to undergo morula compaction. Thus, a lack of UCHL3 may reduce the rate of normal fertilization in IVF. However, this event was not noted in ICSI, which may be due to artificial selection.

The level of a protein was not necessarily consistent with its activity; therefore, the UCH activity was explored. A decrease in the enzymatic activities of UCH was demonstrated in spermatozoa from A or OA. The activity of UCHL3 was the highest among UCHs. UCHL3 was 200times more enzymatically active than UCHL1 [26] and mainly expressed in spermatids [15]. This suggested that the enzymatic activity of UCHL3 may also be decreased in spermatozoa from A or OA. The enzymatic activity of UCH was positively correlated with sperm count, concentration, TM, and PR. Furthermore, there were linear relationships between the UCH enzymatic activity and FR, percentage of embryos suitable for transfer/cryopreservation and high-quality embryo rate in IVF. However, no positive correlations were found among the UCH enzymatic activity, FR, percentage of embryos suitable for transfer/cryopreservation, the rate of high-quality embryos in ICSI, and cleavage rate in both IVF and ICSI. Previous data suggested that the UCH activity was present on the acrosome surface of boar spermatozoa and that high UCHL3 enzymatic activity was measured in a motile spermatozoa fraction with intact acrosomes; UCHL3 was involved in antipolyspermy defense during porcine fertilization, which can be alleviated by the addition of recombinant UCHL3 to the fertilization medium [19]. These results indicated that the decreased activity of UCHL3 may alter sperm acrosome function, which may explain why FR and the percentage of embryos suitable for transfer/cryopreservation were decreased for spermatozoa from A. This study demonstrated negligible effects of UCHL3 on the rates of cleavage and high-quality embryo which should be explored in following trials. Thus, active UCHL3 were needed for the structural and functional integrity of spermatozoa.

UPS plays a critical role in the progression of spermatogenesis. DUBs are involved in spermatogenesis and male fertility, e.g., USP2/UBP-testis, USP8/mUBPy and USP14 [9]. UCHL3 was responsible for the disassembly of the ubiquitin-protein complex or polyubiquitin chains during the substrate priming for proteasomal proteolysis to prevent inappropriate protein degradation [12]. The inhibition of deubiquitination can increase the rate of substrate proteolysis [27]. When both the level and activity of UCHL3 were decreased, the removal of multi-ubiquitin chains from the substrate failed. The present data demonstrated that ubiquitin aldehyde can inhibit the activity of UCHL3. Therefore, ubiquitin aldehyde may modulate the protein expression and function of UCHL3.

A decreased level and activity of UCHL3 in spermatozoa may alter mitochondrial structure and apoptosis and, ultimately, result in a decrease of motility, count, concentration of spermatozoa, and rates of fertilization and embryos suitable for transfer/cryopreservation. These findings suggested that UCHL3 may be a novel regulator for male fertility. This verdict should be verified in following trials involving many more cases. Identification of UCHL3 substrates will be necessary to explore the function and molecular mechanism of UCHL3 underlying sperm characteristics and function.

## Conclusions

The present study demonstrated positive correlations between the level and activity of UCHL3 and sperm characteristics and function. UCHL3 can be used as an indicator of sperm quality, fertilization and early embryo development in patients with asthenozoospermia and oligoasthenozoospermia.

## References

[1] Curi SM, Ariagno JI, Chenlo PH, Mendeluk GR, Pugliese MN, Sardi Segovia LM, et al Asthenozoospermia: analysis of a large population. Arch Androl. 2003; 49(5): 343–9. 12893510 10.1080/01485010390219656

[2] Practice Committee of American Society for Reproductive Medicine in collaboration with Society for Male Reproduction and Urology. Evaluation of the azoospermic male. Fertil Steril. 2008; 90(5 Suppl): S74–7. 10.1016/j.fertnstert.2008.08.092 19007652

[3] Howes L, Jones R. Interactions between zona pellucida glycoproteins and sperm proacrosin/acrosin during fertilization. J Reprod Immunol.2002; 53(1–2): 181–92. 11730915 10.1016/s0165-0378(01)00101-2

[4] Baarends WM, vander-Laan R, Grootegoed JA. Specific aspects of the ubiquitin system in spermatogenesis. J Endocrinol Invest.2000; 23(9): 597–604. 10.1007/BF03343782 11079455

[5] Sutovsky P, Aarabi M, Miranda-Vizuete A, Oko R. Negative biomarker based male fertility evaluation: Sperm phenotypes associated with molecular-level anomalies. Asian J Androl. 2015; 17(4): 554–60. 10.4103/1008-682X.153847 25999356 PMC4492044

[6] Haraguchi CM, Mabuchi T, Hirata S, Shoda T, Tokumoto T, Hoshi K, et al Possible function of caudal nuclear pocket: Degradation of nucleoproteins by ubiquitin-proteasome system in rat spermatids and human sperm. J Histochem Cytochem.2007; 55(6): 585–95. 10.1369/jhc.6A7136.2007 17312012

[7] Sutovsky P. Ubiquitin-dependent proteolysis in mammalian spermatogenesis, fertilization, and sperm quality control: killing three birds with one stone. Microsc Res Tech.2003; 61(1): 88–102. 10.1002/jemt.10319 12672125

[8] Eletr ZM, Wilkinson KD. Regulation of proteolysis by human deubiquitinating enzymes. Biochim Biophys Acta.2014; 1843(1): 114–28. 10.1016/j.bbamcr.2013.06.027 23845989 PMC3833951

[9] Suresh B, Lee J, Hong SH, Kim KS, Ramakrishna S. The role of deubiquitinating enzymes in spermatogenesis. Cell Mol Life Sci.2015; 72(24): 4711–20. 10.1007/s00018-015-2030-z 26350476 PMC11113867

[10] Berruti G, Martegani E. The deubiquitinating enzyme mUBPy interacts with the sperm-specific molecular chaperone MSJ-1: the relation with the proteasome, acrosome, and centrosome in mouse male germ cells. Biol Reprod. 2005; 72(1): 14–21. 10.1095/biolreprod.104.030866 15342353

[11] Gnesutta N, Ceriani M, Innocenti M, Mauri I, Zippel R, Sturani E, et al, Cloning and characterization of mouse UBPy, a deubiquitinating enzyme that interacts with the ras guanine nucleotide exchange factor CDC25(Mm)/Ras-GRF1. J Biol Chem. 2001; 276(42): 39448–54. 10.1074/jbc.M103454200 11500497

[12] Wilkinson KD. Ubiquitination and deubiquitination: targeting of proteins for degradation by the proteasome. Semin Cell Dev Biol.2000; 11(3): 141–48. 10.1006/scdb.2000.0164 10906270

[13] Bose R, Manku G, Culty M, Wing SS. Ubiquitin-proteasome system in spermatogenesis. Adv Exp Med Biol. 2014; 759: 181–213. 10.1007/978-1-4939-0817-2_9 25030765

[14] Kurihara LJ, Semenova E, Levorse JM, Tilghman SM. Expression and functional analysis of Uch-L3 during mouse development. Mol Cell Biol.2000; 20(7): 2498–504. 10713173 10.1128/mcb.20.7.2498-2504.2000 PMC85452

[15] Kwon J, Wang YL, Setsuie R, Sekiguchi S, Sakurai M, Sato Y, et al Developmental regulation of ubiquitin C-terminal hydrolase isozyme expression during spermatogenesis in mice. Biol Reprod. 2004; 71(2): 515–21. 10.1095/biolreprod.104.027565 15084487

[16] Kwon J, Wang YL, Setsuie R, Sekiguchi S, Sato Y, Sakurai M, et al Two closely related ubiquitin C-terminal hydrolase isozymes function as reciprocal modulators of germ cell apoptosis in cryptorchid testis. Am J Pathol. 2004; 165(4): 1367–74. 10.1016/S0002-9440(10)63394-9 15466400 PMC1618639

[17] World Health Organization. WHO laboratory manual for the examination and processing of human semen fifth ed. Geneva: WHO Press; 2010.

[18] Wang MJ, Ou JX, Chen GW, Wu JP, Shi HJ, O WS, et al Does prohibitin expression regulate sperm mitochondrial membrane potential, sperm motility and male fertility? Antioxid Redox Signal. 2012; 17(3): 513–9. 10.1089/ars.2012.4514 22324369

[19] Yi YJ, Manandhar G, Sutovsky M, Li R, Jonáková V, Oko R, et al Ubiquitin C-terminal hydrolase-activity is involved in sperm acrosomal function and anti-polyspermy defense during porcine fertilization. Biol Reprod. 2007; 77(5): 780–93. 10.1095/biolreprod.107.061275 17671268

[20] Bradford MM. A rapid and sensitive method for the quantitation of microgram quantities of protein utilizing the principle of protein-dye binding. Anal Biochem. 1976; 72: 248–54. 942051 10.1016/0003-2697(76)90527-3

[21] Chen H, Wang W, Mo Y, Ma Y, Ouyang N, Li R, et al Women with high telomerase activity in luteinised granulosa cells have a higher pregnancy rate during in vitro fertilization treatment. J Assist Reprod Genet. 2011; 28(9): 797–807. 10.1007/s10815-011-9600-2 21717175 PMC3169683

[22] Sano Y, Furuta A, Setsuie R, Kikuchi H, Wang YL, Sakurai M, et al Photoreceptor cell apoptosis in the retinal degeneration of Uchl3-deficient mice. Am J Pathol. 2006; 169(1): 132–41. 10.2353/ajpath.2006.060085 16816367 PMC1698765

[23] Aitken RJ. Free radicals, lipid peroxidation and sperm function. Reprod Fertil Dev. 1995; 7(4): 659–68. 10.1071/rd9950659 8711202

[24] Kwon J. The new function of two ubiquitin C-terminal hydrolase isozymes as reciprocal modulators of germ cell apoptosis. Exp Anim. 2007; 56(2): 71–7. 17460351 10.1538/expanim.56.71

[25] Mtango NR, Sutovsky M, Susor A, Zhong Z, Latham KE, Sutovsky P. Essential role of maternal UCHL1 and UCHL3 in fertilization and preimplantation embryo development. J Cell Physiol. 2012; 227(4): 1592–603. 10.1002/jcp.22876 21678411 PMC4351554

[26] Liu Y, Fallon L, Lashuel HA, Liu Z, Lansbury PJ. The UCH-L1 gene encodes two opposing enzymatic activities that affect alpha-synuclein degradation and Parkinson's disease susceptibility. Cell. 2002; 111(2): 209–18. 12408865 10.1016/s0092-8674(02)01012-7

[27] Guterman A, Glickman MH. Deubiquitinating enzymes are IN/ (trinsic to proteasome function). Curr Protein Pept Sci. 2004; 5(3): 201–11. 15188770 10.2174/1389203043379756

